# Sequential Inoculation of Indigenous *Starmerella bacillaris* and *Saccharomyces cerevisiae* to Modulate the Volatile Profiles and Lower Ethanol Yields in Romanian Aromatic Wines

**DOI:** 10.3390/foods15162789

**Published:** 2026-08-08

**Authors:** Raluca-Ștefania Rădoi-Encea, Camelia-Filofteia Diguță, Iuliana-Diana Bărbulescu, Diana-Ionela Popescu (Stegăruș), Răzvan-Ionuț Teodorescu, Alexandru-Dumitru Ilie, Florentina Matei

**Affiliations:** 1 Faculty of Food Industry and Tourism, Transilvania University of Brașov, 148 Castelului St., 500014 Brașov, Romania; raluca.encea@unitbv.ro; 2 Faculty of Biotechnologies, University of Agronomic Sciences and Veterinary Medicine Bucharest, 59 Mărăști Blvd., District 1, 011464 Bucharest, Romania; diana.barbulescu@bth.usamv.ro; 3 National Research and Development Institute for Cryogenic and Isotopic Technologies—ICSI Râmnicu Vâlcea, 4th Uzinei St., 240050 Râmnicu Vâlcea, Romania; diana.stegarus@icsi.ro; 4 Faculty of Land Reclamation and Environmental Engineering, University of Agronomic Sciences and Veterinary Medicine Bucharest, 011464 Bucharest, Romania; razvan.teodorescu@usamv.ro; 5 Pietroasa-Istrița Research and Development Center for Viticulture and Pomiculture, 127470 Buzău, Romania; alexandru-dumitru.ilie@pietroasaveche.ro

**Keywords:** indigenous yeasts, wine fermentation, wine typicity

## Abstract

The industrial use of non-*Saccharomyces* yeasts offers a robust bioprocess strategy for modulating volatile biochemical profiles and reducing climate-driven ethanol production in fermented beverages. This study explores the metabolic diversity of indigenous non-*Saccharomyces*, with a particular focus on *Starmerella bacillaris* MI151, selected for sequential inoculation with *Saccharomyces cerevisiae* MI118. Pilot-scale bioreactor fermentations (25 L) of two Romanian matrix-specific aromatic grape cultivars, Busuioacă de Bohotin and Tămâioasă Românească, exhibited clear strain-dependent carbon flux changes. The sequential culture actively triggered the glycerol-pyruvic pathway, which consistently reduced the final ethanol concentration to up to 0.94% (*v*/*v*) and maintained a balanced profile of volatile compounds. GC-MS-based metabolomic profiling revealed substantial changes in the esterification kinetics and glycosidic precursor cleavage. The sequential fermentation resulted in a synergistic increase in *n*-hexyl acetate (up to 2310.01 µg/L) and bypassing of the standard enzymatic repression, freeing highly volatile monoterpenes (β-citronellol and β-geraniol) in the analyzed matrices. This targeted microbial system successfully modulated the organic acid–phenolic balance, neutralizing harsh structural finishes, as confirmed by quantitative sensory mapping. Finally, these specific local yeast strains exhibit strong bioprocess scalability, offering a predictable, non-engineered strategy to lower the ethanol yield while driving targeted flavor enhancement in climate-vulnerable viticultural regions.

## 1. Introduction

Commercial *Saccharomyces cerevisiae* starter cultures ensure consistent fermentation but can limit sensory diversity in wines [1,2]. Recent market trends highlight an increasing consumer preference for wines with distinct regional identity [3,4]. While abiotic factors have traditionally defined terroir, microbial communities contribute to wine typicity by influencing its chemical composition [5,6,7,8]. Within these communities, yeasts, especially indigenous *Saccharomyces cerevisiae*, play a key role in fermentation and sensory attributes [9,10,11]. Additionally, several non-*Saccharomyces* species, such as *Starmerella bacillaris*, *Metschnikowia pulcherrima* and *Torulaspora delbrueckii*, among others, are recognized for their metabolic activities that shape wine aroma, particularly during the early stages of fermentation [12,13,14,15,16,17,18,19,20,21,22]. At the strain level, variability further modulates fermentation performance and aroma production [23,24]. Yeast strains are evaluated according to oenological criteria developed for *S. cerevisiae* and subsequently extended to non-*Saccharomyces* species. Technological traits, including carbon assimilation, tolerance to osmotic and ethanol stress, resistance to acidic conditions and SO_2_, and preservation capacity, ensure fermentation efficiency. Qualitative traits influence wine composition and sensory properties, including the production of aroma-active compounds, reduced formation of undesirable metabolites, and enzymatic activity [19,23,24,25,26,27,28,29]. The role of native yeast populations in defining wine typicity has led to the concept of “microbial terroir”, highlighting the need to preserve indigenous microbiota [6,8,30]. In this context, mixed or sequential fermentations pairing non-*Saccharomyces* species with *S. cerevisiae* represent an effective strategy to enhance wine complexity while maintaining fermentation reliability [14,15,16,21,22,31,32,33].

Romania ranks among Europe’s top ten wine producers, with an expected output of approximately 4.1 million hectoliters in 2025, representing a 29% increase from 2024 [34]. As the global wine industry becomes more competitive, it is important to focus on strategies that enhance the authenticity and quality of Romanian wines [35,36,37,38]. In recent years, the Pietroasa vineyard, a traditional Romanian wine region, has focused on producing wines that express regional typicity, particularly through their sensory profile [39,40,41,42]. However, the diversity and technological properties of indigenous yeast strains from the Pietroasa vineyard remain insufficiently characterized at the strain level, particularly for pilot-scale validation and aroma modulation [41,43,44].

While various non-*Saccharomyces* yeasts have been explored for alcohol reduction, retaining the delicate sensory profiles of terpene-dependent cultivars is an essential bottleneck in metabolic modulation [45]. *Starmerella bacillaris* was established as a potent modifier of wine aroma profiles, characterized by high glycerol production and robust extracellular enzymatic activities capable of hydrolyzing bound volatile precursors [46]. However, the success of sequential inoculation strategies relies heavily on the metabolic compatibility between specific yeast strains and the unique biochemical matrices of distinct grape cultivars. To date, the exploitation of native, co-adapted consortia to manage alcohol production while simultaneously improving the varietal typicity of highly aromatic musts has not been fully realized. While sequential fermentations with *St. bacillaris* have been documented over the last decade, the strain-dependent mechanisms in specific terpene-rich Muscat-type matrices remain insufficiently analyzed. The present study addresses this gap by assessing how native, co-adapted yeast strains selectively release volatile monoterpenes without inducing elevated volatile acidity in climate-inflated sugar musts.

To achieve this, 19 indigenous yeast strains, previously isolated from the Pietroasa vineyard and selected based on their persistence in spontaneous regional fermentations, were evaluated using molecular techniques and oenological screening to identify potential starter cultures for a targeted sequential bioprocess utilizing the indigenous fructophilic strain *Starmerella bacillaris* MI151 in combination with *Saccharomyces cerevisiae* MI118. The present research targeted two distinct Muscat-family cultivars native to Romania, Busuioacă de Bohotin and Tămâioasă Românească, which are highly prized for their intense terpene-driven aromatic profiles but increasingly vulnerable to climate-driven sugar inflation [36]. We hypothesized that utilizing a localized *St. bacillaris* consortium would initiate a natural carbon-flux diversion to reduce final ethanol concentrations while employing its specialized enzymatic machinery to unlock and enrich the signature volatile profiles of such aromatic varieties. Finally, this study establishes a tailored, cultivar-specific microbial strategy to address the paired challenges of alcohol management and aroma optimization in premium winemaking. By utilizing native, co-adapted consortia (*St. bacillaris* MI151 and *S. cerevisiae* MI118) isolated from the same ecological niches as the musts, we expect enhanced metabolic compatibility and a more synchronized exploitation of the particular chemical matrices’ characteristics of regional Muscat-family grapes.

## 2. Materials and Methods

### 2.1. Yeast Strains

A total of nineteen yeast strains (designated MI100, MI102–MI118, MI150, MI151), isolated from the Pietroasa vineyard, Buzău County, Romania, were investigated. In addition, one commercial strain, *Saccharomyces cerevisiae* Lalvin EC-1118 (Lallemand, Montreal, QC, Canada), was included as a reference culture. Cultures stored in the Microorganisms Collection of UASVM B (Bucharest, Romania) at −20 °C in 30% (*v*/*v*) glycerol-YPD broth (Scharlau, Barcelona, Spain) were reactivated on YEPD agar at 25 °C for 24 h.

### 2.2. Yeast Molecular Identification by PCR ITS-RFLP, and Sequencing

Yeast identification was performed using a molecular approach based on Esteve-Zarzoso et al. [47]. Genomic DNA was extracted from 72 h cultures grown in YEPD broth at 25 °C using the Quick-DNA™ Fungal/Bacterial Miniprep Kit (Zymo Research, Irvine, CA, USA), following the manufacturer’s protocol. DNA purity and concentration were assessed using a SpectraMax^®^ QuickDrop™ Micro-Volume Spectrophotometer (Molecular Devices, San Jose, CA, USA).

The ITS region, including the 5.8S rDNA sequence, was amplified using universal primer pair ITS1 and ITS4 [48]. Each 50 µL PCR reaction contained 10× DreamTaq Green Buffer with 20 mM MgCl_2_, 0.5 µM of each primer, 0.2 mM dNTPs, 0.25 U of DreamTaq DNA Polymerase (Thermo Fisher Scientific Baltics, UAB, Vilnius, Lithuania), and MilliQ water to reach the final volume. PCR amplification was performed in a MyCycler thermal cycler (Bio-Rad, Hercules, CA, USA), following the conditions described by Esteve-Zarzoso et al. [47].

PCR amplicons were individually digested with *Hae*III, *Hha*I, and *Hinf*I enzymes (Thermo Fisher Scientific Baltics, UAB, Vilnius, Lithuania), following the manufacturer’s instructions. Amplicons and restriction fragments were separated on 2% agarose gels, and visualized under UV light using a GelDoc-It system (Analytik Jena, Upland, CA, USA), and fragment sizes were estimated with a 100 bp DNA ladder. RFLP profiles were compared to reference patterns in the Yeast-id database (https://www.yeast-id.org/ (accessed on 30 January 2026)).

Representative strains were chosen for Sanger sequencing at the Cellular and Molecular Immunological Applications Center (CEMIA, Larissa, Greece). Species identification was confirmed using BLASTn searches against the NCBI database (https://www.ncbi.nlm.nih.gov/BLAST (accessed on 20 February 2026)), and the obtained sequences were uploaded to GenBank (https://www.ncbi.nlm.nih.gov/genbank (accessed on 8 March 2026)).

### 2.3. In Vitro Screening of Oenological Traits

#### 2.3.1. Carbon Assimilation, Osmotic Tolerance

Carbon assimilation and osmotic stress tolerance were assessed using Yeast Nitrogen Base (YNB) (Sigma-Aldrich, St. Louis, MO, USA) with 2% (*w*/*v*) agar, following Vrinceanu et al. [29]. Assimilation was tested using 2% (*w*/*v*) glucose, fructose, maltose, or sucrose, while osmotic tolerance was evaluated under 30% (*w*/*v*) glucose. Plates were incubated at 25 °C for 72 h, with visible growth being considered a positive assimilation or osmotic tolerance trait.

#### 2.3.2. Stress Tolerance

Tolerance to ethanol and sulfur dioxide was evaluated using a modified protocol based on Sidari et al. [24]. Standardized yeast cell suspensions were streaked onto YEPD agar containing ethanol at final concentrations of 5%, 10%, or 15% (*v*/*v*), or supplemented with potassium metabisulfite to reach 100, 200, 300, and 400 mg/L SO_2_. The pH of all media was adjusted to 4.0 before inoculation. Plates were sealed with Parafilm^®^ and incubated at 25 °C for 48 h. YEPD agar without stress agents served as the control. Growth was visually assessed after incubation and recorded as positive or negative.

Acid tolerance was assessed according to the method of Vrînceanu et al. [29]. Standardized fresh yeast suspensions (OD600 = 0.10 ± 0.01) were inoculated into YEPD broth pre-adjusted to pH 3.0 using 1N HCl. Cultures were incubated at 25 °C for 24 h, after which acid tolerance was evaluated by measuring the final OD600.

#### 2.3.3. Enzymatic Activities

Enzymatic activities relevant to oenology were evaluated on modified media by streaking fresh yeast cultures.

Catalase activity was determined by suspending fresh yeast biomass in a small volume of 3% hydrogen peroxide. Immediate bubble formation was considered a positive result [24].

Phytase activity was evaluated using a culture medium containing 10 g/L peptone, 5 g/L yeast extract, 5 g/L glucose, 5 g/L sodium phytate, 2 g/L magnesium sulfate, 1 g/L calcium chloride, and 20 g/L agar. The appearance of a clear halo around the colonies confirmed phytase activity [49].

Protease activity was tested on Yeast Nitrogen Base (YNB) without amino acids (Sigma-Aldrich, St. Louis, MO, USA). After sterilization, 10% (*w*/*v*) skim milk was added before plating. Clear halos after 5 days at 25 °C revealed proteolytic activity [29].

#### 2.3.4. Acetic Acid and Hydrogen Sulfide Production

Acetic acid production was tested on Chalk agar containing 10 g/L glucose, 3 g/L yeast extract, 3 g/L calcium carbonate, and 15 g/L agar. A clear halo surrounding the colonies indicated acid production [24].

H_2_S production was measured on BiGGY agar plates (Scharlab S.L., Barcelona, Spain) after 48 h at 25 °C. Colony colors, ranging from white to dark brown or black, indicated increasing levels of H_2_S production [25].

#### 2.3.5. Killer Phenotype

The killer/sensitive phenotype was assessed following the work of Matei & Găgeanu [50] using an adapted medium containing 20 g/L glucose, 10 g/L peptone, 10 g/L yeast extract, 0.3 g/L methylene blue, and 20 g/L agar in sodium citrate buffer (pH 4.8). *Saccharomyces cerevisiae* 17/17 served as the sensitive strain, and SMR4 as the killer reference. Reference strains were spread onto plates, and test isolates were streaked over them. Plates were incubated at 18 °C for 72 h. Blue zones confirmed sensitivity, blue zones with inhibition halos showed killer activity, and no reaction was considered neutral.

#### 2.3.6. Yeast Strain Preservation by Freeze-Drying

Long-term preservation of yeast isolates was performed by lyophilization, as described by Vrinceanu et al. [29]. Cells from 24 h cultures were harvested by centrifugation (2000× *g* for 5 min at 4 °C), washed twice with phosphate-buffered saline and resuspended in 2 mL of sterile 5% (*w*/*v*) glucose solution (PanReac AppliChem, Darmstadt, Germany) as a cryoprotectant. Suspensions were pre-frozen overnight at −20 °C and subsequently freeze-dried in a FreeZone 6 system (Labconco, Kansas City, MO, USA) at −55 °C and 0.3 mbar for 4 h. Survival rate was calculated as follows:

$$\%\;viability=\frac{log\;CFU\;Nfd}{log\;CFU\;Ni}\times 100$$

where CFU Ni and CFU Nfd represent viable counts before and after freeze-drying, respectively.

### 2.4. Winemaking Potential—Pilot-Scale Fermentations

To demonstrate the winemaking potential of local yeast strains, pilot-scale fermentations were conducted. Two indigenous aromatic grape varieties, pink variety Busuioacă de Bohotin (coded BB) and white variety Tămâioasă Românească (coded TR), both from the Pietroasa vineyard in Buzău County, were used. Based on their oenological properties, two indigenous yeast strains (*St. bacillaris* MI151 and *S. cerevisiae* MI118) were selected for pilot-scale fermentations.

#### 2.4.1. Experimental Variants

Three fermentation variants were evaluated. Wines BB-St and TR-St were fermented with *St. bacillaris*, while BB-Sc and TR-Sc were fermented with *S. cerevisiae* MI118. Wines BB-St/Sc and TR-St/Sc were produced by sequential fermentation, with *S. cerevisiae* being inoculated 48 h after the initial inoculation of *St. bacillaris*. Yeast metabolic activity and successful sequential interaction were confirmed through finished wine volatile profiling and physico-chemical footprinting rather than direct viable cell counting.

#### 2.4.2. Winemaking Technology

Grapes were harvested at technological maturity and processed by destemming and crushing to obtain the must. At the beginning of processing, potassium metabisulfite was added at a dose corresponding to 50 mg/L total SO_2_, alongside a commercial pectolytic enzyme preparation containing polygalacturonase (PG, 4500 U/g) and pectin lyase (PL, 130 U/g) at a dosage of 0.4 mL/100 kg of grapes. Maceration protocols differed between the two grape varieties. The must from Tămâioasă Românească grapes was cooled to 10 °C and subjected to an extended maceration for 20 h. In contrast, for Busuioacă de Bohotin, the must and skins were transferred to a maceration tank pre-set to 10 °C; once filled, the liquid underwent 2 h of recirculation, using the tank’s integrated pump system without moving the solid pomace mass. This targeted recirculation ensured controlled contact between the must and skins while avoiding aggressive extraction. Throughout the maceration process, the must remained in contact with the grape skins to promote the extraction of varietal aroma compounds and other skin-derived constituents characteristic of each grape variety. The initial Tămâioasă Românească must contained approximately 225 g/L, a pH of 3.3, and a total acidity was 5.5 g/L tartaric acid, with a nitrogen profile of 122 mg/L ammonia and 124 mg/L Primary Amino Nitrogen (PAN). The Busuioacă de Bohotin must contained approximately 235 g/L sugar, a pH of 3.3, total acidity of 5.8 g/L tartaric acid, 153 mg/L ammonia and 86 mg/L PAN.

Before inoculation, the musts were filtered to eliminate indigenous microflora and to prevent interference with the tested yeast strains. Fresh yeast cultures (10^6^ CFU/mL) were then inoculated into each batch, to ensure rapid fermentation onset. Fermentations were conducted in 25 L stainless steel tanks, with three independent replicates being performed for each treatment. Fermentation progress was monitored daily by measuring density. Alcoholic fermentation was considered complete upon reaching technological dryness, defined as a stable density and a residual sugar concentration of ≤1 g/L. Final residual sugar levels were quantitatively verified by laboratory analysis. Alcoholic fermentations lasted approximately 2–3 weeks, with fermentation temperatures maintained at 14–16 °C for Tămâioasă Românească and 17–19 °C for Busuioacă de Bohotin. Following alcoholic fermentation, both experimental variants underwent a similar conditioning and stabilization process. The wines were racked off the gross lees immediately after sedimentation had occurred and were sulfited with 60 mg/L SO_2_, together with the addition of 1 g/L bentonite for clarification and protein stabilization. After 2 weeks, the wines were filtered using SDL 195 sterile filter sheets (<0.6 µM) and stored in tanks at 4 °C for at least 2 months prior to primary analytical evaluations. Low-temperature storage was maintained to promote tartaric stabilization, preserve volatile aroma compounds, and minimize oxidative processes. Finally, the wines were filtered through SDL 197 filter sheets (<0.45 µM) and bottled in 750 mL glass bottles sealed with natural corks.

#### 2.4.3. Physico-Chemical Analyses

The ethanol content was measured following the OIV-MA-AS312-01 method (Part D, Type IV Method), which involves measuring the refractive index of light passing through the distillates obtained from the wine samples. The alcohol by volume (% *v*/*v*) was determined based on the refractive index at 20 °C as specified in the Compendium of the International Organization of Vine and Wine [51].

Commercial enzymatic and colorimetric kits (Biosystems, Barcelona, Spain) were used to determine glucose, fructose, total acidity, lactic acid, sulfites, and polyphenols, with an automatic photometric analyzer (BioSystems Y15, Biosystems, Spain). Glucose and fructose concentrations were determined using a D-glucose/D-fructose kit (hexokinase/PGI method) and expressed in g/L (limit of detection, LOD: 0.02 g/L; limit of linearity, LOL: 8.00 g/L; repeatability CV ≤ 1.4%, within-laboratory CV ≤ 2.8%). Total acidity was measured using a Total Acidity kit (bromothymol blue method), calibrated with aqueous primary standards, and expressed as g/L of tartaric acid (measurement interval: 1.6–16.5 g/L; repeatability CV ≤ 1.9%, within-laboratory CV ≤ 4.0%). Lactic acid concentrations were determined using a L-Lactic acid kit (L-lactate dehydrogenase method), calibrated with a Multiacid Standard kit, and expressed in g/L (LOD: 0.02 g/L; LOL: 3.00 g/L; repeatability CV ≤ 2.3%, within-laboratory CV ≤ 3.8%). Free sulfite content was determined using a Free Sulfite kit based on the pararosaniline method (LOD: 0.7 mg/L; LOL: 100 mg/L; repeatability CV ≤ 1.3%, within laboratory CV ≤ 2.1%). Total sulfite content was determined using a Total Sulfite kit based on 5,5′-dithio-2-nitrobenzoic acid reaction (LOD: 3 mg/L; LOL: 400 mg/L; repeatability CV ≤ 1.9%, within-laboratory CV ≤ 2.3%). Both free and total sulfites were expressed in mg/L. Total polyphenols were measured using a Polyphenols kit (Folin–Ciocâlteu method) calibrated with a gallic acid standard (2000 mg/L) and expressed as mg/L of gallic acid equivalents (LOD: 60 mg/L; LO: 3000 mg/L; repeatability CV ≤ 2.2%, within-laboratory CV ≤ 2.81%). The pH values were measured using a Mettler Toledo pH meter (Columbus, OH, USA). All analytical methodologies applied were in accordance with the standard guidelines established by the International Organisation of Vine and Wine (OIV).

The aroma profiles of the wine samples were analyzed by GC–MS following storage at 4.0 ± 0.5 °C. Volatile compounds were extracted by liquid–liquid microextraction (LLME). For sample extraction, an 8 mL aliquot of wine was mixed with 2.5 g NaCl and 4 mL dichloromethane (DCM). The mixture was vortexed (2500 rpm, 3 min), cooled in an ice bath (5 min), and centrifuged (3000 rpm, 3 min). 2 mL consisting of the organic layer was collected, dried over 0.5 g anhydrous Na_2_SO_4_, re-centrifuged under identical conditions, and filtered through a 0.45 µm PTFE membrane. Extractions were performed in five replicates to ensure analytical precision. Chromatographic separation was conducted using a triple-quadrupole GC–MS system (Agilent Technologies, Santa Clara, CA, USA) equipped with an Rtx-5MS column (30 m × 0.25 mm × 0.25 µm). Extracts (1 µL) were injected in splitless mode, at a temperature of 220 °C. Helium served as the carrier gas at a constant flow rate of 1.4 mL/min. The oven temperature program was configured as follows: initial temperature of 40 °C (held for 7 min), increased to 95 °C at 5 °C/min (held for 4 min), then increased to 120 °C at 5 °C/min (held for 10 min), and finally increased to 280 °C at 55 °C/min (held for 15 min). Mass spectra were acquired in electron ionization (EI) mode at 70 eV, over an *m*/*z* range of 35–350. Compound identification was based on spectral matching with the NIST MS Search 2.4 database (National Institute of Standards and Technology, Gaithersburg, MD, USA). For quantification, 29 reference standards (Sigma-Aldrich, Darmstadt, Germany) representing alcohols, esters, aldehydes, acids, and terpenoids were used. Stock solutions of 1000 mg/L and 10 mg/L were serially diluted to produce calibration levels of 5–500 µg/L and 2–50 mg/L. Each level was injected in triplicate and quantified by external matrix-matched calibration. Recovery was evaluated using simulated wine (distilled water + 12.5% ethanol + 2 mg/L standards) processed under identical LLME conditions. Extraction recovery rates were determined by spiking wine samples with known amounts of target standards. The calculated recovery rates were then used to correct GC–MS data for both extraction efficiency and the 2:1 concentration ratio (8 mL wine sample volume to 4 mL DCM extraction solvent). This empirical recovery correction method ensured accurate quantification without introducing potential chromatographic co-elution risks associated with exogenous internal standards. The results are expressed in µg/L.

#### 2.4.4. Sensory Analysis of the Local Wines

Wine samples were presented in randomized order and served at the appropriate tasting temperature in coded glasses. The sensory characteristics of the experimental wines were evaluated by a panel of 22 trained examiners, who regularly participate in such sessions. The panel comprised 60% women and 40% men, all aged 25 to 50. The tasting took place at the Pietroasa Viticulture and Winemaking Research and Development Station—SDCVV Pietroasa, UASVM Bucharest (Buzău, Romania), in accordance with ISO 8589:2007, ISO 3591:1997, and OIV guidelines [52,53,54], and was conducted without endangering the participants. The examiners assessed appearance (clarity and color intensity), olfactory characteristics (aroma strength), gustatory perception (alcohol, acidity, astringency, body, and finish), and overall wine quality using a Descriptive Analysis (DA) on a structured 10-point intensity scale, where 1 indicated weak or absent sensations and 10 very strong sensations. They also described the wines’ color, aroma notes (fruity, floral, spicy), sugar perception, and other aromas. All participants in this local wine tasting session were informed of the confidentiality policy, gave their consent to participate, and agreed to the use of their results for this study.

### 2.5. Statistical Analysis

All experiments were performed in triplicate, with data expressed as mean ± standard deviation (SD). Normality of distribution was assessed using the Shapiro–Wilk test. Significant differences among wine samples were evaluated using one-way analysis of variance (ANOVA) followed by Tukey’s post hoc test, while the Mann–Whitney test was applied when appropriate. Statistical analyses and data visualization were performed using Microsoft Excel 365 and XLSTAT 2025 add-in (Addinsoft, New York, NY, USA) [55].

## 3. Results and Discussion

### 3.1. Molecular Identification of Yeast Isolates

In this study, PCR-ITS-RFLP analysis generated highly discriminative restriction patterns, enabling clear species-level identification of the analyzed yeast isolates (Table 1). The restriction patterns were species-specific and corresponded to *Pichia* species (*P. kudriavzevii* and *P. membranifaciens*), *Starmerella bacillaris*, *Metschnikowia pulcherrima*, *Candida railenensis*, *Hanseniaspora* species (*H. uvarum* and *H. valbyensis*), *Torulaspora delbrueckii*, *Wickerhamomyces anomalus*, and *Saccharomyces cerevisiae*.

The 5.8S–ITS region is widely used as a molecular marker for the rapid identification of yeast isolates and allows for reliable species-level discrimination within indigenous populations [44,48,56]. Representative ITS sequences were submitted to NCBI databases, with accession numbers ranging from PZ120327 to PZ120336 (Table 2), thereby providing molecular confirmation of their taxonomic classification.

The identified yeast community, comprising *Saccharomyces* and non-*Saccharomyces* species, represents a characteristic microbial fingerprint of the Pietroasa vineyard, consistent with previous studies [41,44,45]. Frequently isolated from winemaking environments, these species are of increasing interest as commercial or non-conventional starter cultures in vinification [12,13,14,16,17,19,21,57].

### 3.2. In Vitro Oenological Characterization of Indigenous Yeast Isolates

Screening of indigenous yeast isolates revealed significant strain-dependent variability in metabolic and stress tolerance traits, highlighting their potential for targeted selection in controlled wine fermentations. Table 3 contains the physiological profiling of the indigenous yeast strains, in terms of sugar assimilation, osmotic tolerance, gas production, tolerance to sulfur dioxide and ethanol, acetic acid and hydrogen sulfide production and killer phenotype.

Glucose assimilation confirms fermentative capacity, while variability in fructose and maltose utilization reflects strain-dependent metabolic differences relevant to fermentation performance. High osmotic tolerance observed in all strains demonstrates strong adaptation to high-sugar must and supports their application in high-gravity fermentations. Gas production was detected only in *S. cerevisiae* strains MI117 and MI118, as well as in the commercial strain EC-1118. The ability of selected strains to assimilate carbon sources and grow under osmotic stress conditions shows their adaptation to high-sugar musts, which is essential for efficient fermentation [15,24,29].

*S. cerevisiae* strains demonstrated higher ethanol tolerance, indicating their ability to complete fermentation. In contrast, non-*Saccharomyces* strains exhibited lower tolerance and are more suitable for early fermentation stages. The variation in ethanol tolerance among the isolates aligns with earlier studies that emphasize strain-specific differences in stress tolerance in native yeasts [15,23,29].

Acetic acid production was generally low among the tested isolates. Nevertheless, higher levels were observed in *W. anomalus* MI111 and *P. membranifaciens* MI112, which may increase volatile acidity and influence wine aroma and balance. Hydrogen sulfide production remained low in strains such as *Candida railenensis* MI150, *Hanseniaspora* spp., and *M. pulcherrima*, which helps prevent sulfur-related off-aromas. These results indicate a lower risk of sulfur-related off-flavors and reduced acetic acid production. The selected isolates are suitable for controlled fermentation processes, consistent with previous studies [15,24,28,29,31].

The killer phenotype was observed in *H. uvarum* MI107 and two *St. bacillaris* strains (MI114 and MI151). Killer activity can give these strains a distinct advantage during fermentation by inhibiting sensitive yeast populations [50,58].

**Figure 1:**
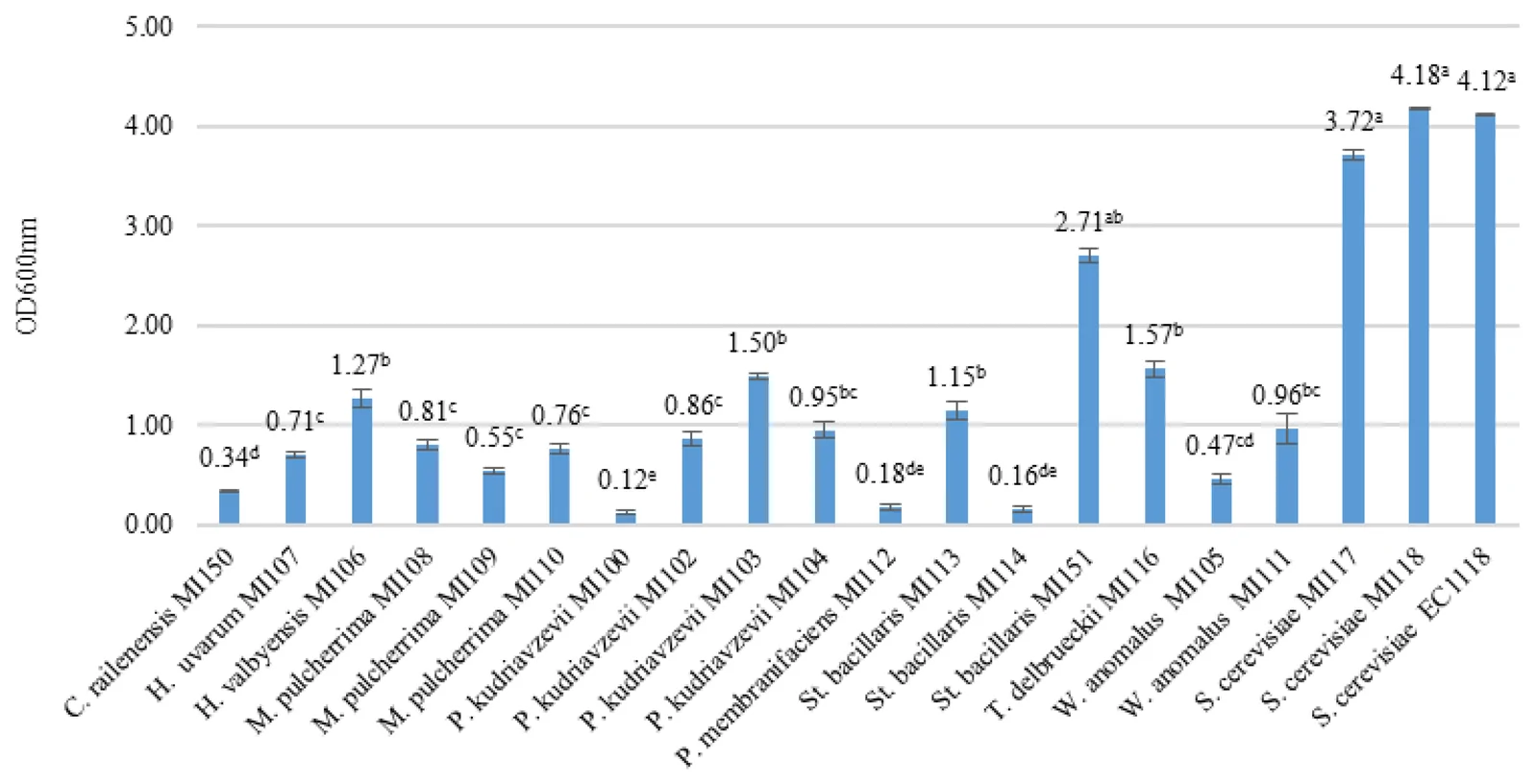
Growth of yeast isolates at pH 3 determined by optical density at 600 nm (OD600). Values represent mean ± standard deviation of three independent experiments (*n* = 3). Different letters highlight significant differences between strains according to one-way ANOVA followed by Tukey’s test (*p* < 0.05).

All isolates also tolerated sulfur dioxide up to 400 mg/L, demonstrating their strong adaptation to winemaking conditions. This resistance is essential for their potential use as starter or co-starter cultures, securing stability and performance during fermentation [24]. Tolerance to acidic conditions varied significantly among the isolates (*p* < 0.05). *S. cerevisiae* strains exhibited the highest tolerance to low pH, maintaining higher growth levels at pH 3 (Figure 1). Also, several non-*Saccharomyces* isolates, particularly *St. bacillaris* MI151 and *T. delbrueckii* MI116, also showed considerable growth, indicating their potential contribution during early fermentation stages under acidic conditions (Figure 1). The resistance to sulfur dioxide and growth in acidic environments demonstrate the extensive ability of indigenous yeasts to adapt to oenological stress factors [24,29].

Enzymatic activities varied across the isolates tested (Table 4). Catalase activity was found in most strains, including *M. pulcherrima, P. kudriavzevii, St. bacillaris, T. delbrueckii, W. anomalus,* and *S. cerevisiae*. Compared to previous studies, the extent of catalase activity indicates a strong adaptation of these indigenous strains to oxidative conditions [59,60]. In contrast, phytase activity was detected in fewer isolates, mainly *H. uvarum*, *St. bacillaris*, and *S. cerevisiae*, supporting its expression at varying levels across species and the limited distribution reported in the literature [49]. Protease activity was observed exclusively in *M. pulcherrima* strains (MI108–MI110), indicating a role in protein breakdown and nitrogen release, which can influence fermentation kinetics and wine stability [15,24,28]. These results highlight the functional diversity among yeast species. They also show their contribution to nutrient transformation and wine quality.

### 3.3. Survival of Indigenous Yeast Strains After Freeze-Drying

**Figure 2:**
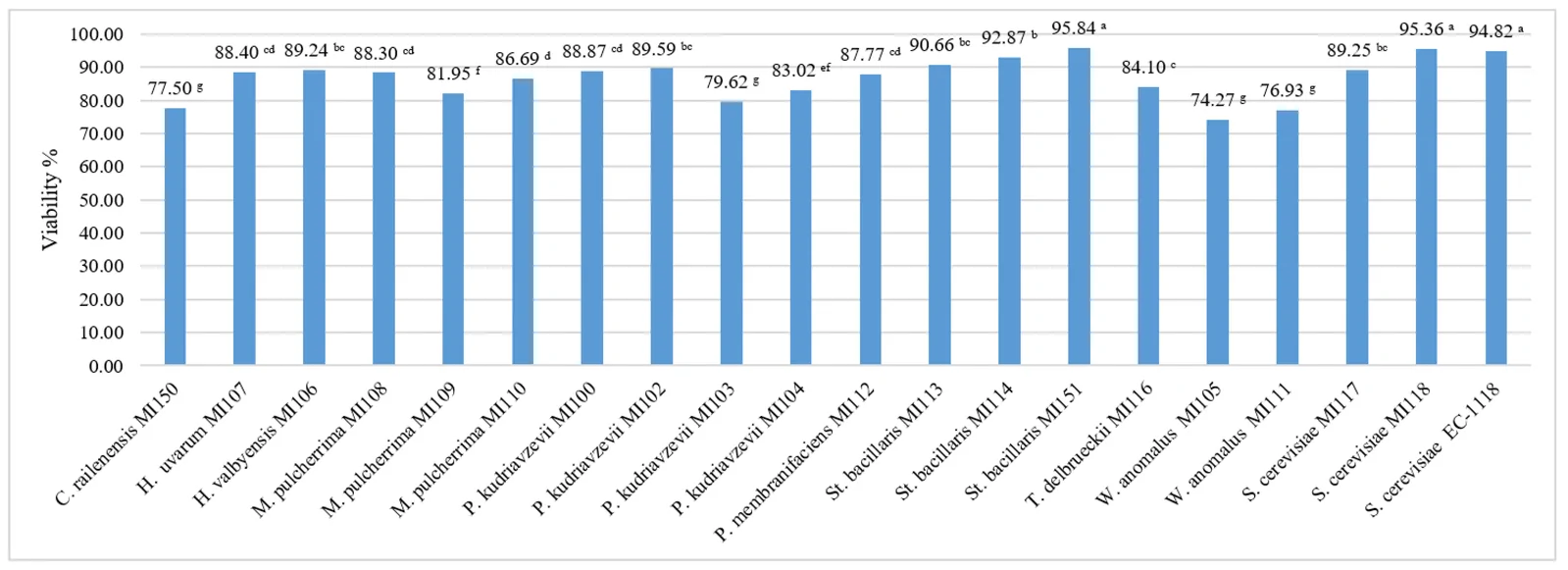
Viability (%) of yeast strains after freeze-drying. Values represent mean ± standard deviation (*n* = 3). Different letters highlight significant differences between strains according to Tukey’s test (*p* < 0.05).

Survival rates after freeze-drying ranged from 74% to 96% (*p* < 0.05) (Figure 2). Survival rates exceeding 90% for selected strains confirm their suitability for industrial starter culture production and support their application in controlled fermentation processes. In contrast, *W. anomalus* and *C. railenensis* had lower survival rates, indicating they may be more sensitive to dehydration stress. These findings are in agreement with reported post-freeze-drying survival rates, often exceeding 80–90% when appropriate cryoprotectants are employed [29,41,59].

### 3.4. Assessment of Indigenous Yeasts Under Pilot-Scale Fermentation Conditions

Following the technological screening, *S. cerevisiae* MI118 and *St. bacillaris* MI151 were chosen for pilot-scale fermentation trials. *S. cerevisiae* MI118 displayed strong fermentation traits, including ethanol tolerance up to 15%, and resistance to sulfur dioxide, which are vital for a reliable alcoholic fermentation. Meanwhile, *St. bacillaris* MI151 demonstrated its tolerance under acidic and sulfite conditions, with moderate hydrogen sulfide (H_2_S) production and killer activity, making it suitable for mixed fermentations. These features justified additional testing both as monocultures and co-cultures.

#### 3.4.1. Physicochemical Characterization of Experimental Wines

The physicochemical composition of the wines demonstrates that yeast selection directly influenced key fermentation parameters, including ethanol production, sugar consumption, acidity, sulfite levels, and polyphenol retention (Table 5).

Ethanol concentrations ranged from 13.36% to 14.50% (*v*/*v*). Wines fermented with *S. cerevisiae* MI118 showed slightly higher ethanol levels compared to those obtained with *St. bacillaris* MI151, while sequential fermentations resulted in lower values, indicating a moderate reduction in ethanol yield under mixed conditions. These values are in agreement with the concentrations reported by Colibaba et al. [39], who found 12.9% (*v*/*v*) ethanol for Busuioacă de Bohotin and 13.6% (*v*/*v*) for Tămâioasă Românească. Comparable ranges were also reported by Englezos et al. [32] (13.11–13.47% *v*/*v*) and Russo et al. [61] (13.67–13.68% *v*/*v*), while slightly higher values (14.6–14.7% *v*/*v*) were observed by Englezos et al. [14,46].

Residual sugars showed substantial variation among samples, highlighting the importance of fermentation efficiency. The two sequential fermentations presented the highest concentrations (~0.54 g/L), while the other variants exhibited lower levels, ranging down to 0.33 g/L, implying near-complete sugar consumption across all fermentation strategies. These values are slightly higher than those reported by Francesca et al. [62] (0.07 g/L) and Russo et al. [61] (0.78–0.85 g/L) but fall below those reported by du Plessis et al. [63] (2.23 g/L), indicating an overall high level of sugar depletion.

Lactic acid remained at trace levels in Busuioacă de Bohotin wines, whereas higher concentrations were observed in Tămâioasă Românească wines (0.34–0.38 g/L), indicating a higher tendency toward secondary fermentation in these variants. Total acidity ranged from 3.70 to 4.80 g/L, with higher values in Busuioacă de Bohotin wines, while pH values ranged from 3.34 to 3.72. These figures fall below those reported by Colibaba et al. [39] (6.36 g/L for Tămâioasă Românească wines and 7.03 g/L for Busuioacă de Bohotin wines) and by Englezos et al. [46] (6.27–8.11 g/L) and Englezos et al. [32] (7.07–8.26 g/L).

Free sulfur dioxide levels ranged from 7 to 21 mg/L, while total sulfur dioxide levels ranged from 70 to 108 mg/L, indicating differences in sulfur dioxide levels under the applied fermentation conditions. The observed values remain lower than those previously observed by Colibaba et al. [39], which were 37.13 mg/L for free SO_2_ and 128.8 mg/L for total SO_2_ in Busuioacă de Bohotin wines, and 46.1 mg/L and 133.6 mg/L for Tămâioasă Românească wines. Francesca et al. [62] reported 17.50 mg/L free and 30.00 mg/L total sulfur dioxide in Frappato wine produced by *S. cerevisiae* and *St. bacillaris* co-fermentation. Nisiotou et al. [33] reported 8.6 and 18.8 mg/L (free and total SO_2_) for co-fermentation, and 13.7 and 26.0 mg/L for sequential fermentation using the same yeasts.

Total polyphenol levels varied from 226 to 368 mg/L, with Busuioacă de Bohotin wines exhibiting higher values than Tămâioasă Românească wines. These results are consistent with the phenolic potential reported by Bărbulescu et al. [36] and Frîncu et al. [42] confirming the influence of grape composition on phenolic content.

#### 3.4.2. Volatile Compound Distribution

The total content of volatile aroma compounds varied significantly among samples, influenced by fermentation strategy and grape variety (*p* < 0.05) (Table 6).

Alcohol levels ranged from 854.35 to 1921.65 µg/L, with the highest values found in BB-Sc and TR-St/Sc samples, while sequential fermentation produced lower concentrations in the Busuioacă de Bohotin wine, consistent with previous findings [33]. This pattern reflects the well-known metabolic characteristics of *Starmerella bacillaris*, whose metabolic pathway is preferentially directed to glycerol production, resulting in a lower ethanol yield than *S. cerevisiae* [46]. The influence of *St. bacillaris* on higher alcohols accumulation is strain-dependent and varies based on the protocol. Some studies report that *S. cerevisiae—St. bacillaris* mixed fermentations yield higher levels of higher alcohols than *S. cerevisiae* single-strain fermentations [32], while others show decreases in isoamyl alcohol and increases in isobutanol [46,64], indicating a compound-specific redistribution of carbon metabolism.

Organic acids showed a distinct pattern, peaking at TR-St/Sc (3782.68 µg/L) and BB-Sc (3062.53 µg/L), with sequential fermentations displaying distinctly reduced levels in the BB-St/Sc (2495.97 µg/L) but the highest overall accumulation in the TR wine, showing matrix- and strain-dependent organic acid modulation during sequential inoculation. Esters exhibited notable differences, with the highest concentrations in BB-St/Sc (3507.91 µg/L) and TR-St/Sc (3284.04 µg/L), demonstrating that sequential fermentations recorded the highest overall ester accumulation. These compounds significantly contribute to fruity and floral aromas, indicating an enhanced enzymatic capacity for ester synthesis under sequential inoculation conditions. Aldehyde concentrations remained low across all variants, ranging from 24.16 to 111.84 µg/L, indicating controlled fermentation conditions and limited oxidative reactions.

Oppositely, terpenoids were substantially higher in Tămâioasă Românească wines, especially in TR-St/Sc (750.23 µg/L), highlighting both the strong varietal influence and the contribution of sequential fermentation on terpene accumulation. Grape variety strongly influenced terpenoid distribution.

Notably, the biochemical shifts show that sequential fermentation strategy achieves a targeted qualitative enhancement, driven by the autochthonous strains’ metabolic modulation and the sensory thresholds of key analytes.

The distribution of individual volatile compounds (Table 7) varied significantly among samples, with both variables as the primary factors (*p* ≤ 0.05).

In this study, the volatile profile comprised 29 compounds, mainly alcohols and esters. Single-strain *S. cerevisiae* and sequential *St. bacillaris/S. cerevisiae* inoculations exhibited higher levels of aroma compounds compared to single-strain *St. bacillaris* fermentations. Across all fermentation strategies, Tămâioasă Românească wines exhibited overall higher concentrations than Busuioacă de Bohotin.

Wines fermented with *S. cerevisiae* showed higher concentrations of key metabolites. The highest values were observed for 2,3-butanediol in BB-Sc (822.36 µg/L) and octanoic acid in BB-Sc (1304.08 µg/L). Capric acid peaked in TR-St/Sc (968.14 µg/L), which may reflect increased metabolic activity related to fatty acid synthesis. Ester compounds showed variability across samples, with ethyl hexanoate reaching 315.11 µg/L in BB-Sc, while ethyl decanoate and ethyl butyrate reached maximum values in TR-St/Sc (286.3 µg/L and 159.12 µg/L, respectively). The high concentration of *n*-hexyl acetate in BB-St/Sc (2310.01 µg/L) and TR-St/Sc (1070.1 µg/L) indicates enhanced fresh and fruity-green notes under sequential fermentation, whereas the higher levels of diethyl succinate in TR wines, particularly TR-St/Sc (614.07 µg/L), suggest a greater contribution to aromatic complexity and sensory roundness. These compounds predominantly contributed to fruity aroma notes in wines fermented either with *S. cerevisiae* MI118 alone or in sequential fermentation with *St. bacillaris*. These results are consistent with previous studies showing that esters and higher alcohols are key contributors to wine aroma [26]. The increase in specific esters under sequential fermentation supports earlier findings that *St. bacillaris* can enhance ethyl ester formation when combined with *S. cerevisiae* [33].

Higher alcohols varied across fermentation strategies, with 2-phenylethanol and 1-hexanol reaching their highest levels in TR-St/Sc (514.07 µg/L and 205.34 µg/L, respectively). These results support the contribution of higher alcohols to floral and green-related notes and highlight the combined influence of yeast interactions and grape-derived precursors in Tămâioasă Românească wines. This pattern is consistent with previous studies reporting enhanced phenylethyl alcohol production in fermentations involving *St. bacillaris* [65], while previous findings similarly demonstrates that mixed or sequential fermentations involving this species can moderate ethanol levels [33]. The elevated 2-phenylethanol in TR-St/Sc wines reflects a likely combined effect: nitrogen consumption by *St. bacillaris* during the initial fermentation phase, which under nitrogen-limiting conditions strongly promotes aromatic alcohol production via the Ehrlich pathway in all yeast strains [66], followed by enhanced synthesis by *S. cerevisiae* as it takes over fermentation. The elevated concentrations found in TR wines compared to BB wines further suggest that the aromatic amino acid precursors of Tămâioasă Românească, a Muscat-family variety characterized by high phenylalanine availability, amplify this metabolic response, since increased aromatic amino acid concentrations have been shown to stimulate 2-phenylethanol production regardless of nitrogen status [66]. The aromatic significance of 2-phenylethanol in wines is well established, as it contributes rose-like, floral, and honey notes and is among the most sensorially impactful higher alcohols produced during [67,68]. The elevated 1-hexanol concentrations in TR-St/Sc (205.34 µg/L) further contribute to green and grassy notes that complement the floral profile of this variety, while 2,3-butanediol reached its highest level in BB-Sc (822.36 µg/L), consistent with the well-documented capacity of *S. cerevisiae* to produce this compound as a by-product of pyruvate metabolism, and in agreement with findings that mixed fermentations with *St. bacillaris* generally yield higher amounts of higher alcohols compared to pure *S. cerevisiae* controls [32].

Fatty acids, including *n*-hexanoic, octanoic, and capric acids, were consistently higher in *S. cerevisiae* fermentations and in Tămâioasă Românească wines. Octanoic acid reached its maximum in TR-St/Sc (1475.36 µg/L), while capric acid also peaked in the same sample (968.14 µg/L), showing an active lipid metabolism and serving as a strong precursor pool for ester synthesis. These results are consistent with the established metabolic profile of *S. cerevisiae*-dominated fermentations, in which *St. bacillaris* single inoculation and sequential inoculation have been reported to decrease fatty acid content relative to *S. cerevisiae* single inoculation, showing the lower lipid metabolic activity of the non-*Saccharomyces* species [64]. The high fatty acid levels observed in TR-St/Sc are particularly noteworthy because octanoic and capric acids serve as direct precursors for the synthesis of their corresponding ethyl esters (ethyl octanoate and ethyl decanoate) which were also elevated in the same sample (180.36 µg/L and 286.3 µg/L, respectively), indicating an active ester-forming pathway stimulated by sequential fermentation metabolism. This esterification of medium-chain fatty acids to ethyl ester has been described as a key mechanism in yeast lipid metabolism during wine fermentation [68]. Volatile fatty acids are produced during yeast metabolism and can influence wine aroma depending on their concentration [69]. The consistently higher fatty acid levels in TR wines across all fermentation strategies further confirm the strong influence of grape variety on the fatty acid precursor pool available for ester synthesis, as previously observed for other aromatic varieties [70].

Terpenoid compounds were strongly influenced by grape variety, with β-linalool and β-geraniol reaching their highest concentrations in TR-St/Sc (167.08 µg/L and 130.04 µg/L), contributing to floral and citrus-like aromas [22]. These results indicate that fruity attributes were mainly associated with ester formation, more pronounced in Busuioacă de Bohotin wines, while terpene-derived floral characteristics dominated in Tămâioasă Românească wines. The dominance of these monoterpenes in TR wines directly reflects the varietal identity of Tămâioasă Românească, which belongs to the Muscat Blanc à Petits Grains family. In Muscat-type cultivars, linalool, geraniol, and citronellol are the predominant monoterpenes, accumulating progressively throughout berry development and perceived by humans at very low olfactory thresholds due to their floral, rose-like, and citrus aromatic character [71]. The terpene profile of Tămâioasă Românească wines in the present study, dominated by β-linalool, β-geraniol, R-α-terpineol, β-citronellol, and eucalyptol, is in full agreement with the characteristic Muscat-type aroma signature of this variety, which is defined by geraniol, linalool, α-terpineol, limonene, geranial, and β-citronellal as its principal volatile markers [22,72]. *St. bacillaris* MI151 successfully uncoupled the release of Muscat-family monoterpenes from conventional ethanol production, optimizing the varietal typicity of both Busuioacă de Bohotin and Tămâioasă Românească matrices, corroborated by a significant release of linalool and geraniol within sequential trials. Terpenes are primarily located in the skin of the grape berry and exist in two forms: free volatile forms that directly contribute to aroma, and odorless precursors that can be released enzymatically during fermentation [71]. The higher terpene concentrations observed in sequential fermentations (TR-St/Sc) compared to single-strain wines suggest that *St. bacillaris*, during its active phase preceding *S. cerevisiae* inoculation, contributes to the hydrolytic release of terpenoid compounds [22,45]. This is further supported by earlier observations that fermentation of aromatic grape musts with *St. bacillaris* produced enhanced accumulation of citronellol and geraniol in the final wines [45]. The detection of eucalyptol exclusively in TR wines, with a notable increase from TR-Sc (77.12 µg/L) to TR-St/Sc (138.4 µg/L), provides additional evidence that sequential fermentation with indigenous *St. bacillaris* reveals the full terpene potential of this aromatic variety. By contrast, terpene accumulation in Busuioacă de Bohotin was limited to R-α-terpineol in BB-St and BB-St/Sc, and β-citronellol in BB-St/Sc, confirming that this variety’s aromatic typicity is expressed primarily through ester-derived fruity notes rather than monoterpene-driven floral notes. This result aligns with the general recognition that terpene expression in wine is regulated by grape cultivar, with Muscat-family varieties consistently producing higher and more diverse terpene profiles than non-aromatic or semi-aromatic varieties across all winemaking conditions [71].

Other compounds, such as furfural and decanal, remained at low concentrations, indicating a limited contribution of oxidative processes.

Overall, single-strain *St. bacillaris* fermentations yielded modest volatile levels, whereas sequential inoculation with *S. cerevisiae* significantly increased their levels, indicating a synergistic effect on aroma profile. Although yeast population dynamics were not directly monitored during the fermentations, the distinct metabolic signatures clearly demonstrate that sequential fermentation strategy had a highly desirable effect of the final wine profiles. Notably, the metabolic activity of *St. bacillaris* MI151 did not lead to an increase in volatile acidity. Acetic acid concentrations remained below the sensory threshold (Table 7), confirming that this specific sequential inoculation strategy successfully avoids the metabolic drawbacks frequently associated with sluggish non-*Saccharomyces* kinetics. While commercial *S. cerevisiae* strains offer fast and highly predictable kinetics, sequential fermentations using indigenous *St. bacillaris* alongside *S. cerevisiae* proved capable of maintaining comparable fermentation rates, while significantly enhancing the overall aromatic complexity and sensory evaluation of both Busuioacă de Bohotin and Tămâioasă Românească wines. Present results confirm that the use of indigenous *St. bacillaris* and *S. cerevisiae* strains from the Pietroasa vineyard, particularly in sequential combination, constitutes an effective strategy for preserving and enhancing the local aromatic typicity of both varieties, in agreement with the empirical findings that support the autochthonous non-conventional yeasts as valuable tools for wine distinction and regional identity [32,33,65].

#### 3.4.3. Sensory Evaluation of Experimental Wines

**Figure 3:**
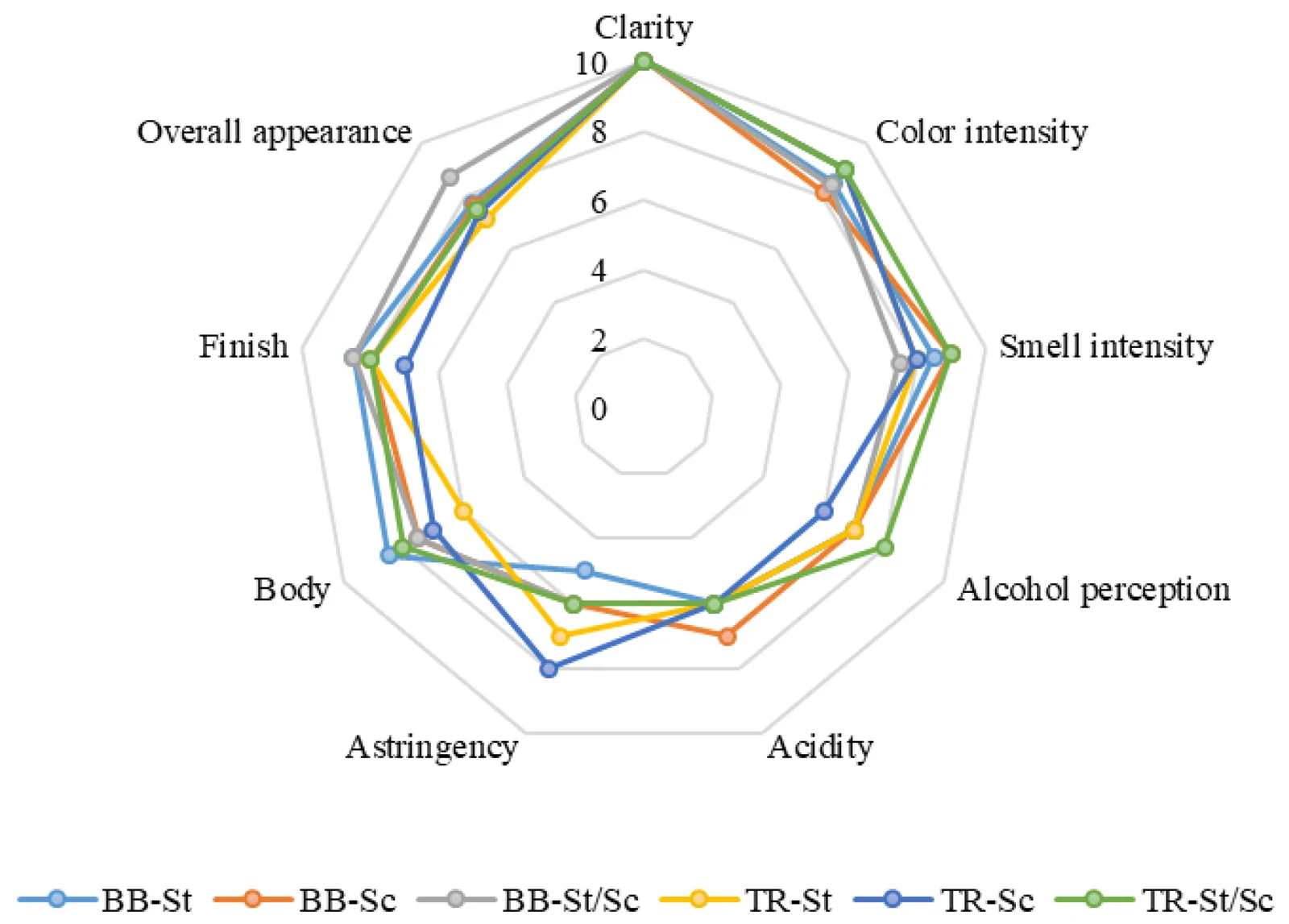
Descriptive sensory analysis of experimental wines (BB-St—Busuioacă de Bohotin fermented by *St. bacillaris* MI151; BB-Sc—Busuioacă de Bohotin fermented by *Saccharomyces cerevisiae* MI118; BB-St/Sc—Busuioacă de Bohotin fermented by *St. bacillaris* MI151 and *S. cerevisiae* MI118; TR-St—Tămâioasă Românească fermented by *St. bacillaris* MI151; TR-Sc—Tămâioasă Românească fermented by *S. cerevisiae* MI118; TR-St/Sc—Tămâioasă Românească fermented by *St. bacillaris* MI151 and *S. cerevisiae* MI118).

Descriptive sensory analysis indicated good overall acceptability for all experimental wines, with scores above 7.0 for both grape varieties (Figure 3). Wine typicity reflects the integration of chemical composition, grape variety, and microbial activity, including yeasts, which contribute to distinct and recognizable sensory profiles [3,4]. The perception of alcohol aligned with the actual ethanol measurements, while perceived acidity varied due to differences in total acidity and pH. The finish was influenced by aroma-active compounds, mainly esters and terpenoids.

These findings emphasize that aroma perception depends on the balance and interactions among compounds, rather than their concentrations. The most used descriptors for Busuioacă de Bohotin wines were “rose”, “red fruit”, “strawberry” and “wild flowers”. BB-St wine was perceived as semi-dry and slightly sour, while BB-Sc and BB-St/Sc wines were perceived as dry. The color of these wines was described differently, ruby for BB-St, rose red for BB-Sc, and orange-red for BB-St/Sc, respectively. For the Tămâioasă Românească wines, the most used descriptors were “tangerine”, “golden apple”, “wild flowers”, “citrus”, “mango” and “banana”. All the three TR wines were perceived as dry and were described as pale yellow.

#### 3.4.4. Multivariate Analysis of Volatile Fingerprints

**Figure 4:**
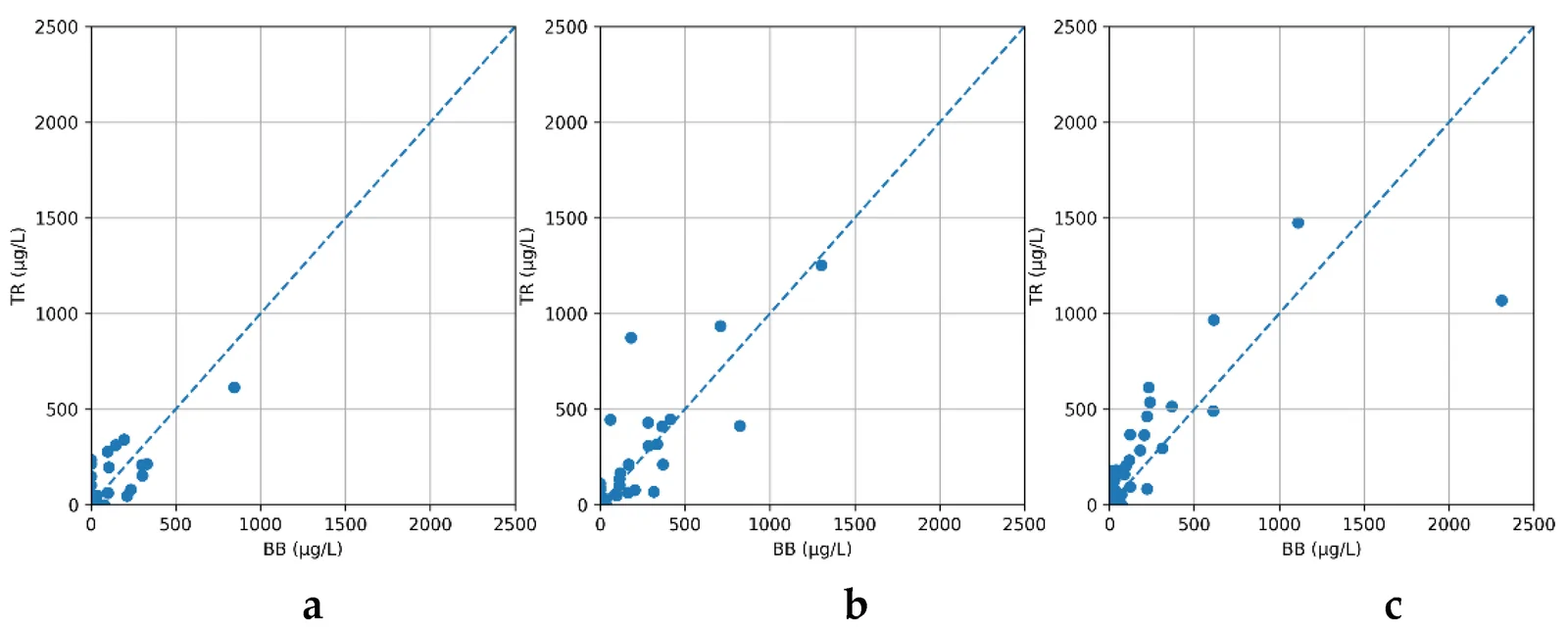
Scatter plots of aroma profile identified in wines produced by different fermentation strategies. Each point represents an individual compound quantified by GC–MS in BB (Busuioacă de Bohotin) and TR (Tămâioasă Românească) wines obtained with *St. bacillaris* MI151 (**a**), with *Saccharomyces cerevisiae* MI118 (**b**), and by sequential fermentation of *St. bacillaris* MI151 and *S. cerevisiae* MI118 (**c**). The diagonal line (y = x) indicates equal concentrations between cultivars.

The volatile compounds distribution was significantly modulated by both the grape variety and the fermentation strategy. Fermentation with *St. bacillaris* alone (Figure 4a) showed moderate data dispersion, consistent with its lower production of aroma compounds compared to *S. cerevisiae*. Wines fermented with *S. cerevisiae* (Figure 4b) exhibited a more concentrated distribution, suggesting a more uniform production of volatile compounds and a stable metabolic profile. In contrast, the sequential fermentation (Figure 4c) displayed a greater compound scatter, indicating greater variability in metabolite levels. These plots show the interactions between yeast species and changes in metabolic flux, which influence the wine’s complexity and aroma profile. The occurrence of extreme values for certain compounds indicates that sequential fermentation selectively promotes certain metabolites rather than uniformly broadening the aroma profile.

The systematic off-diagonal deviations across all samples confirm a strong varietal influence, critical for understanding how fermentation conditions shape aroma complexity and sensory perception, demonstrating that grape composition influences compound distribution. These relationships provide a basis for selecting fermentation strategies to target specific aroma profiles.

The fermentation trials were conducted on a pilot scale, with mono- and sequential fermentations being evaluated. The present study provides a basis for further research into co-inoculation strategies, metabolomics, and microbial interactions. This study was based on a single vintage, and seasonal variability was not assessed.

## 4. Conclusions

This study characterized 19 indigenous yeast strains from the Pietroasa vineyard, highlighting significant strain-dependent variations in important technological traits across both *Saccharomyces* and non-*Saccharomyces* species. The application of a targeted pilot-scale bioprocess strategy, using *S. cerevisiae* MI118 and *St. bacillaris* MI151, showed potential in addressing the challenges of climate-driven ethanol production and aroma dilution. In sequential fermentations of the terpene-rich matrices of Busuioacă de Bohotin and Tămâioasă Românească, this inoculation strategy successfully lowered the final ethanol concentrations compared to the single-strain *S. cerevisiae* controls. Volatile and oenological analyses indicated a redistribution of aroma compounds, where the metabolic and enzymatic profile of the indigenous *St. bacillaris* strain optimized the monoterpenes release. This process preserved the sensory profile of these Muscat-family cultivars and prevented the accumulation of volatile acidity, while sensory differences remained limited.

These findings suggest the strong potential of this non-engineered strategy for using indigenous yeasts as local starter cultures to enhance wine authenticity, predictability, and regional typicity in premium Pietroasa wines. Future research should assess strain performance under industrial conditions and across vintages, with a focus on strain–matrix interactions and culture stability during repeated fermentations.

## Figures and Tables

**Table 1:** Species identification of yeast isolates by PCR–RFLP analysis of the ITS region.

Strain Code	PCR Products	Restriction Fragments’ Length			Species Identification
		*Hha*I	*Hinf*I	*Hae*III	
MI150	650	280 + 280	300 + 300	400 + 130 + 80	*Candida railenensis*
MI106	775	650 + 110	240 + 210 + 170 + 130	775	*Hanseniaspora valbyensis*
MI107	775	340 + 320 + 100	370 + 200 + 160	775	*Hanseniaspora uvarum*
MI108; MI109; MI110	400	205 + 100 + 95	200 + 190	280 + 100	*Metschnikowia pulcherrima*
MI100; MI102; MI103; MI104	510	210 + 180 + 70	220 + 150 + 130	380 + 90	*Pichia kudriavzevii*
MI112	480	160 + 110 + 80 + 60	280 + 200	330 + 90 + 50	*Pichia membranifaciens*
MI113; MI114; MI151	460	200 + 100 + 100 + 60	230 + 230	460	*Starmerella bacillaris*
MI116	800	330 + 220 + 150 + 100	410 + 380	800	*Torulaspora delbrueckii*
MI105; MI111	650	580	310 + 310	600 + 50	*Wickerhamomyces anomalus*
MI117; MI118; MI119	840	385 + 365 + 155	365 + 155	320 + 230 + 180 + 150	*Saccharomyces cerevisiae*

**Table 2:** Accession number of yeast strains.

Yeast Strains	Accession Number
*Candida railenensis* MI150	PZ120327
*Hanseniaspora uvarum* MI107	PZ120328
*Hanseniaspora valbyensis* MI106	PZ120329
*Metschnikowia pulcherrima* MI110	PZ120330
*Pichia kudriavzevii* MI104	PZ120331
*Pichia membranifaciens* MI112	PZ120332
*Starmerella bacillaris* MI151	PZ120333
*Torulaspora delbrueckii* MI116	PZ120334
*Wickerhamomyces anomalus* MI111	PZ120335
*Saccharomyces cerevisiae* MI118	PZ120336

**Table 3:** Functional properties of yeast strains.

Yeast Strains	Carbon Assimilation ^a^				Osmotic Tolerance ^a^	Gas Production ^b^	SO_2_ Resistance ^a^				Ethanol Tolerance ^a^				Acetic Acid Production ^c^	H_2_S Production ^d^	Killer Phenotype ^e^	
	Glucose	Fructose	Maltose	Sucrose	30% Glucose		100 mg/L	200 mg/L	300 mg/L	400 mg/L	5%	10%	12%	15%			17/17	SMR4
*C. railenensis* MI150	+	+	+	+	+	−	+	+	+	+	+	+	+	±	−	white	−	−
*H. uvarum* MI107	+	+	−	+	+	−	+	+	+	+	+	−	−	−	−	tan	+	+
*H. valbyensis* MI106	+	+	+	+	+	−	+	+	+	+	+	−	−	−	−	tan	−	−
*M. pulcherrima* MI108	+	+	+	+	+	−	+	+	+	+	+	+	+	±	−	tan	−	−
*M. pulcherrima* MI109	+	+	+	+	+	−	+	+	+	+	+	+	+	−	−	tan	−	−
*M. pulcherrima* MI110	+	+	+	+	+	−	+	+	+	+	+	+	+	±	−	tan	−	−
*P. kudriavzevii* MI100	+	+	+	+	+	−	+	+	+	+	+	+	+	±	−	dark brown	−	−
*P. kudriavzevii* MI102	+	+	+	+	+	−	+	+	+	+	+	+	+	−	−	dark brown	−	−
*P. kudriavzevii* MI103	+	+	+	+	+	−	+	+	+	+	+	+	+	±	−	dark brown	−	−
*P. kudriavzevii* MI104	+	+	+	+	+	−	+	+	+	+	+	+	+	±	−	dark brown	−	−
*P. membranifaciens* MI112	+	+	+	+	+	−	+	+	+	+	+	+	−	−	++	dark brown	−	−
*St. bacillaris* MI113	+	±	+	+	+	−	+	+	+	+	+	−	−	−	−	dark brown	−	+
*St. bacillaris* MI114	+	±	−	+	+	−	+	+	+	+	+	+	−	−	+	dark brown	+	−
*St. bacillaris* MI151	+	+	+	+	+	−	+	+	+	+	+	+	±	−	++	dark tan	+	−
*T. delbrueckii* MI116	+	+	+	+	+	−	+	+	+	+	+	+	+	−	−	dark brown	−	−
*W. anomalus* MI105	+	+	+	+	+	−	+	+	+	+	+	+	+	±	−	dark brown	−	−
*W. anomalus* MI111	+	+	+	+	+	−	+	+	+	+	+	+	+	±	++	dark brown	−	−
*S. cerevisiae* MI117	+	+	+	+	+	+	+	+	+	+	+	+	+	+	+	dark brown	−	−
*S. cerevisiae* MI118	+	+	+	+	+	+	+	+	+	+	+	+	+	+	+	dark tan	−	−
*S. cerevisiae* EC-1118	+	+	+	+	+	+	+	+	+	+	+	+	+	+	+	dark brown	−	−

Legend: ^a^ Growth after 1–2 days of incubation: − no growth, ± uncertain growth, + biomass developed; ^b^ Bubble formation: − no bubbles, + bubbles observed; ^c^ Halo formation: − none detected, + halo detected, ++ larger halo detected; ^d^ Biomass color from white to dark; ^e^ Killer phenotype: − neutral phenotype, + killer or sensitive phenotype.

**Table 4:** Enzymatic activities of yeast isolates relevant to oenology.

Title 1	Enzymatic Activity		
	Catalase	Phytases	Proteases
*C. railenensis* MI150	−	−	−
*H. uvarum* MI107	−	+	−
*H. valbyensis* MI106	+	−	−
*M. pulcherrima* MI108	+	−	+
*M. pulcherrima* MI109	+	−	+
*M. pulcherrima* MI110	+	−	+
*P. kudriavzevii* MI 100	+	−	−
*P. kudriavzevii* MI102	+	−	−
*P. kudriavzevii* MI 103	−	−	−
*P. kudriavzevii* MI 104	+	−	−
*P. membranifaciens* MI112	−	−	−
*St. bacillaris* MI113	+	−	−
*St. bacillaris* MI114	+	+	−
*St. bacillaris* MI151	−	+	−
*T. delbrueckii* MI116	+	−	−
*W. anomalus* MI 105	−	−	−
*W. anomalus* MI111	+	−	−
*S. cerevisiae* MI117	+	+	−
*S. cerevisiae* MI118	+	+	−
*S. cerevisiae* EC-1118	+	+	−

Legend: + enzymatic activity detected; − no enzymatic activity detected.

**Table 5:** Physicochemical characteristics of wines produced after alcoholic fermentation.

Physico-Chemical Analysis	BB-St	BB-Sc	BB-St/Sc	TR-St	TR-Sc	TR-St/Sc
Ethanol content % (*v/v*)	13.98 ± 0.12 ^bc^	14.20 ± 0.09 ^b^	13.86 ± 0.11 ^c^	13.36 ± 0.08 ^d^	14.50 ± 0.16 ^a^	13.56 ± 0.07 ^d^
Glucose and fructose (g/L)	0.33 ± 0.004 ^d^	0.47 ± 0.005 ^b^	0.54 ± 0.08 ^a^	0.37 ± 0.01 ^c^	0.46 ± 0.01 ^b^	0.54 ± 0.08 ^a^
Lactic acid (g/L)	0.03 ± 0.001 ^c^	0.03 ± 0.001 ^c^	0.05 ± 0.001 ^c^	0.38 ± 0.01 ^a^	0.38 ± 0.01 ^a^	0.34 ± 0.01 ^b^
Total acidity (g/L)	4.80 ± 0.07 ^a^	4.40 ± 0.06 ^b^	4.30 ± 0.04 ^bc^	4.20 ± 0.04 ^c^	3.70 ± 0.06 ^c^	3.70 ± 0.04 ^d^
pH	3.34 ± 0.03 ^d^	3.54 ± 0.04 ^c^	3.59 ± 0.04 ^bc^	3.52 ± 0.02 ^c^	3.72 ± 0.02 ^a^	3.66 ± 0.02 ^ab^
Free sulfur (mg/L)	21.00 ± 0.30 ^a^	12.00 ± 0.17 ^b^	9.00 ± 0.14 ^c^	12.00 ± 0.10 ^b^	10.00 ± 0.15 ^c^	7.00 ± 0.12 ^e^
Total sulfur (mg/L)	108.00 ± 1.15 ^a^	76.00 ± 0.70 ^d^	83.00 ± 0.75 ^c^	87.00 ± 1.04 ^b^	70.00 ± 0.27 ^e^	82.00 ± 0.95 ^c^
Polyphenols (mg/L)	279.00 ± 2.88 ^c^	368.00 ± 4.37 ^a^	301.03 ± 2.26 ^b^	226.00 ± 2.74 ^d^	298.00 ± 3.75 ^b^	288.00 ± 4.07 ^c^

All values are presented as mean (*n* = 3) ± standard deviation (SD). Different superscript letters within the same row highlight significant differences between samples (Tukey’s test, *p* ≤ 0.05).

**Table 6:** Total content of aroma compounds in the experimental wines after alcoholic fermentation.

Aromatic Compounds (µg/L)	Wine Samples					
	BB-St	BB-Sc	BB-St/Sc	TR-St	TR-Sc	TR-St/Sc
Alcohols	1008.64 ± 50.84 ^a^	1921.65 ± 164.50 ^c^	1340.27 ± 95.08 ^a^	854.35 ± 36.82 ^a^	1423.86 ± 92.29 ^ab^	1717.59 ± 128.18 ^bc^
Organic acids	1394.99 ± 47.26 ^c^	3062.53 ± 94.78 ^b^	2495.97 ± 86.79 ^a^	1575.79 ± 108.05 ^c^	3274.84 ± 176.01 ^b^	3782.68 ± 217.95 ^b^
Esters	554.45 ± 17.60 ^a^	1516.47 ± 114.84 ^b^	3507.91 ± 55.40 ^d^	890.81 ± 126.22 ^a^	2311.01 ± 125.14 ^c^	3284.04 ± 186.80 ^d^
Aldehydes	66.83 ± 2.32 ^a^	66.08 ± 1.56 ^a^	99.82 ± 2.26 ^b^	26.19 ± 1.06 ^a^	24.16 ± 1.06 ^a^	111.84 ± 6.17 ^c^
Terpenoids	116.50 ± 15.48 ^a^	157.12 ± 9.68 ^a^	126.74 ± 6.66 ^a^	173.22 ± 9.91 ^a^	495.74 ± 16.45 ^b^	750.23 ± 31.24 ^c^

All values are presented as mean (*n* = 3) ± standard deviation (SD). Different letters in superscript on the same row highlight significant differences between the wine samples (Tukey’s post hoc test, *p* ≤ 0.05).

**Table 7:** Strain-driven modulation of individual aroma compounds in Busuioacă de Bohotin and Tămâioasă Românească wines determined by GC–MS.

Aromatic Compounds (µg/L)	Wine Samples					
	BB-St	BB-Sc	BB-St/Sc	TR-St	TR-Sc	TR-St/Sc
**Esters**						
Methyl isovalerate	45.25 ± 3.05 ^a^	96.33 ± 9.14 ^c^	69.15 ± 1.97 ^b^	nd	50.11 ± 3.08 ^a^	53.11 ± 2.14 ^a^
Ethyl butyrate	nd	113.00 ± 8.89 ^b^	88.15 ± 11.42 ^a^	nd	104.9 ± 6.45 ^ab^	159.12 ± 2.12 ^c^
Ethyl hexanoate	nd	315.11 ± 110.04 ^d^	222.05 ± 21.96 ^c^	40.12 ± 2.11 ^a^	70.11 ± 2.36 ^b^	84.15 ± 4.65 ^b^
Ethyl octanoate	nd	30.11 ± 1.06 ^a^	nd	nd	nd	180.36 ± 15.12 ^b^
Ethyl decanoate	nd	164.11 ± 9.71 ^c^	180.01 ± 22.41 ^c^	37.3 ± 3.05 ^a^	64.8 ± 4.18 ^b^	286.3 ± 16.42 ^c^
2-Phenethyl acetate	101.78 ± 7.68 ^a^	166.41 ± 9.78 ^c^	115.25 ± 10.22 ^ab^	63.14 ± 4.14 ^a^	212.05 ± 13.14 ^d^	233.18 ± 22.09 ^d^
*n*-Hexyl acetate	212.15 ± 10.97 ^a^	180.70 ± 10.11 ^a^	2310.01 ± 29.14 ^d^	48.3 ± 3.17 ^a^	875.12 ± 93.22 ^b^	1070.1 ± 158.11 ^c^
Diethyl succinate	nd	60.15 ± 5.19 ^a^	231.05 ± 24.17 ^b^	236.47 ± 13.15 ^b^	445.29 ± 43.19 ^b^	614.07 ± 56.58 ^c^
Methyl-4-hydroxy butanoate	195.27 ± 11.01 ^a^	284.01 ± 22.18 ^c^	204.12 ± 17.80 ^ab^	341.37 ± 125.14 ^cd^	310.13 ± 68.56 ^bc^	366.51 ± 74.21 ^d^
**Higher alcohols**						
2-Phenylethanol	331.08 ± 31.14 ^a^	412.37 ± 35.01 ^b^	366.65 ± 22.14 ^ab^	214.07 ± 23.41 ^a^	448.37 ± 74.30 ^bc^	514.07 ± 75.7 ^c^
1-Hexanol	105.14 ± 7.06 ^a^	117.67 ± 9.14 ^ab^	96.14 ± 5.05 ^a^	197.14 ± 16.3 ^c^	167.14 ± 4.18 ^bc^	205.34 ± 18.2 ^c^
2-Pentanol	234.14 ± 22.07 ^c^	204.09 ± 20.19 ^bc^	122.14 ± 29.25 ^b^	80.14 ± 6.14 ^a^	77.15 ± 14.11 ^a^	94.55 ± 15.13 ^ab^
*n*-Propanol	nd	336.14 ± 73.84 ^b^	121.11 ± 8.67 ^a^	104.14 ± 7.03 ^a^	317.12 ± 13.04 ^b^	368.04 ± 72.19 ^c^
Benzyl alcohol	36.14 ± 31.54 ^ab^	29.02 ± 1.02 ^a^	22.15 ± 1.15 ^a^	48.76 ± 3.36 ^b^	nd	45.28 ± 5.04 ^b^
2,3-Butanediol	302.14 ± 9.13 ^a^	822.36 ± 141.05 ^d^	612.08 ± 87.12 ^c^	210.1 ± 21.07 ^a^	414.08 ± 51.12 ^b^	490.31 ± 70.05 ^bc^
**Fatty acids**						
*n*-Hexanoic acid	98.25 ± 9.95 ^a^	281.50 ± 16.03 ^c^	220.11 ± 14.11 ^b^	278.5 ± 26.11 ^b^	430.7 ± 53.07 ^b^	463.3 ± 96.14 ^b^
Octanoic acid	845.14 ± 45.14 ^a^	1304.08 ± 66.58 ^c^	1111.05 ± 70.14 ^b^	614.14 ± 85.11 ^a^	1254.11 ± 73.18 ^c^	1475.36 ± 76.12 ^d^
Capric acid	nd	709.06 ± 60.12 ^b^	614.25 ± 47.19 ^b^	214.4 ± 12.19 ^a^	936.07 ± 148.07 ^c^	968.14 ± 149.36 ^c^
Butyric acid	nd	34.04 ± 2.12 ^a^	nd	nd	29.43 ± 5.05 ^a^	42.07 ± 3.04 ^b^
Isovaleric acid	304.40 ± 5.13 ^b^	369.44 ± 12.05 ^b^	312.09 ± 4.12 ^b^	154.5 ± 18.13 ^a^	212.36 ± 4.01 ^a^	297.34 ± 26.3 ^b^
Malic acid	147.2 ± 8.36 ^a^	364.41 ± 23.07 ^c^	238.47 ± 13.06 ^b^	314.25 ± 57.12 ^bc^	412.17 ± 29.31 ^b^	536.47 ± 97.11 ^c^
**Terpenoids**						
β-Linalool	nd	nd	nd	150.11 ± 9.67 ^b^	113.17 ± 12.07 ^a^	167.08 ± 10.12 ^c^
β-Citronellol	nd	nd	30.70 ± 3.64 ^a^	nd	30.04 ± 2.17 ^a^	80.03 ± 9.36 ^b^
β-Geraniol	nd	nd	28.10 ± 2.01 ^a^	nd	92.13 ± 6.12 ^b^	130.04 ± 9.12 ^c^
R-α-Terpineol	80.36 ± 15.04 ^b^	114.07 ± 9.14 ^c^	38.80 ± 4.16 ^a^	nd	136.27 ± 8.33 ^c^	181.21 ± 13.17 ^d^
Eucalyptol	nd	nd	nd	23.11 ± 2.18 ^a^	77.12 ± 2.08 ^b^	138.4 ± 22.69 ^c^
**Other compounds**						
Furfural	30.34 ± 1.09 ^ab^	31.08 ± 1.09 ^b^	28.22 ± 1.08 ^ab^	26.19 ± 1.06 ^a^	24.16 ± 1.06 ^a^	27.7 ± 1.08 ^ab^
Decanal	36.49 ± 2.05 ^a^	nd	nd	nd	nd	84.14 ± 6.07 ^b^
Acetal	nd	35.00 ± 1.12 ^a^	71.60 ± 1.99 ^b^	nd	nd	nd

All values are presented as mean (*n* = 3) ± standard deviation (SD). Different letters in superscript on the same row highlight significant differences between the wine samples (Tukey’s post hoc test, *p* ≤ 0.05).

## Data Availability

The original contributions presented in this study are included in this article. Further inquiries can be directed to the corresponding authors.
